# Perceiving blocks of emotional pictures and sounds: effects on physiological variables

**DOI:** 10.3389/fnhum.2013.00295

**Published:** 2013-06-21

**Authors:** Anne-Marie Brouwer, Nelleke van Wouwe, Christian Mühl, Jan van Erp, Alexander Toet

**Affiliations:** ^1^Department of Perceptual and Cognitive SystemsTNO, Soesterberg, Netherlands; ^2^Department of Neurology, Vanderbilt UniversityNashville, TN, USA; ^3^INRIA Bordeaux Sud-OuestTalence Cedex, France

**Keywords:** valence, arousal, heart rate, skin conductance, sensory modality

## Abstract

Most studies on physiological effects of emotion-inducing images and sounds examine stimulus locked variables reflecting a state of at most a few seconds. We here aimed to induce longer lasting emotional states using blocks of repetitive visual, auditory, and bimodal stimuli corresponding to specific valence and arousal levels. The duration of these blocks enabled us to reliably measure heart rate variability as a possible indicator of arousal. In addition, heart rate and skin conductance were determined without taking stimulus timing into account. Heart rate was higher for pleasant and low arousal stimuli compared to unpleasant and high arousal stimuli. Heart rate variability and skin conductance increased with arousal. Effects of valence and arousal on cardiovascular measures habituated or remained the same over 2-min intervals whereas the arousal effect on skin conductance increased. We did not find any effect of stimulus modality. Our results indicate that blocks of images and sounds of specific valence and arousal levels consistently influence different physiological parameters. These parameters need not be stimulus locked. We found no evidence for differences in emotion induction between visual and auditory stimuli, nor did we find bimodal stimuli to be more potent than unimodal stimuli. The latter could be (partly) due to the fact that our bimodal stimuli were not optimally congruent.

## Introduction

Identifying an individual's emotions through (neuro)physiological correlates is desirable in a wide range of situations. Examples are continuous and non-interfering evaluation of products like software (Hazlett and Benedek, 2007), improving communication between humans and computers (Picard, 1997) and monitoring patients suffering from phobia or anxiety or trainees in virtual reality environments (Lang et al., 1998; Repetto et al., 2009; Brouwer et al., 2011).

Even though studies on autonomic responses to emotion reported heterogeneous results (Cacioppo et al., 2000; Kreibig, 2010), in an extensive review of the literature Kreibig (2010) makes the case that recordings from the autonomic nervous system can indeed inform us about specific experienced emotions. According to Stemmler (2004) this is to be expected because emotions have distinct goals which require specific sympathetic “fight or flight” and parasympathetic “rest and digest” autonomic responses in order to prepare the body for the appropriate actions. A number of other models at varying conceptual levels have been proposed to clarify the link between emotion and physiological responses (see for a comprehensive review Kreibig, 2011). All of these models view emotions as the instigator of the physiological changes that in turn adapt the organism to (planned) action.

When setting out to study physiological correlates of emotions, one needs to induce emotion in the experimental participant. A relatively straightforward way to affect emotional state is by showing emotion inducing pictures. The International Affective Picture System (IAPS) has been developed in 1988 (Lang et al., 1988) and since then used in numerous studies. Each picture in this database was rated by large groups of participants for arousal (ranging from calm to excited), valence (ranging from pleasant to unpleasant) and dominance (ranging from in control to being dominated). Almost all studies investigating physiological responses to emotion inducing pictures use stimulus locked variables; variables that are defined and measured with respect to the moment that the stimulus appeared. For example, heart rate and skin conductance are aligned to image onset to analyze acceleration or peaks, respectively. These variables describe states of a few seconds after stimulus onset. However, in many situations where one would like to measure (neuro)physiological correlates of emotion such as those described in the first paragraph, there are no clear stimuli to lock responses to, or it would be impractical to relate (neuro)physiological variables to specific stimuli. Also, measuring emotional states over longer periods of time than a few seconds would be desirable. A final advantage of measuring stimulus-unlocked physiological responses over longer intervals is that certain potentially informative physiological variables cannot be reliably determined over a few seconds. In particular, intervals of minimally 1 minute are necessary to determine (high frequency) heart rate variability meaningfully (Task Force, 1996; Berntson et al., 1997). An exception to studies using physiological responses locked to emotional stimuli is a study by Baumgartner et al. (2006a). They induced three different emotional states (happiness, sadness, and fear) by using 70-s sequences of emotional pictures and/or 70-s classical musical excerpts. Heart rate, skin conductance, and respiration variables were found to reflect these three emotional states to some extent. In the current study we aim to induce emotional states by presenting stimuli of specific valence and arousal levels in blocks. We examine heart rate variability, heart rate and skin conductance without using information about stimulus onset. A potential difficulty of using blocks of discrete stimuli to induce emotional states is that observers' responses (emotion) may habituate. Therefore we also examine potential effects of valence and arousal over time. A study by Bradley et al. (1996), in which blocks of unpleasant, pleasant, and neutral images were presented, indicates that at least for stimulus locked measures effects of valence (possibly confounded by arousal) remained constant over time for skin conductance and heart rate, and even increased for facial electromyography.

Like visual stimuli, auditory stimuli can be used to induce emotional states. As mentioned above, Baumgartner et al. (2006a) did not only use pictures, but also musical excerpts to induce happiness, sadness, and fear. They compared subjective and physiological variables between modalities. While involvement was higher for music than for pictures, as reflected by subjective involvement ratings and physiological arousal measures, subjectively reported experienced emotion overlapped better with the intended emotion for pictures than for music. Stimulus material was not chosen in order to specifically vary levels of arousal or valence which perhaps led to few effects of emotion on neurophysiological measures (reduced skin conductance responses for happy compared to sad and fear conditions, and increased respiration rate for fear and happiness compared to sadness when music was involved). Modality did not affect the physiological distinguishability of the three emotions except for the mentioned interaction effect on respiration rate indicating a larger difference in respiration rate between emotions as evoked by music than pictures. Music differs from pictures in more than modality or modality-related aspects. One difference is that a clear inherent meaning as associated with pictures lacks for music. This may explain that emotion as intended by music in Baumgartner et al. (2006a) was less well recognized for music than for pictures. Analogue to the IAPS, Bradley and Lang (2000) developed the International Affective Digitized Sounds (IADS) database that contains acoustic stimuli rated for arousal, valence, and dominance. These stimuli are relatively short in duration and while some of them are (very short) musical excerpts, the large majority of these sounds are associated with inherent meaning (e.g., a gun shot or the sound of a cheering crowd). Bradley and Lang (2000) found that acoustic stimuli from the IADS produced qualitatively similar physiological reactions as those elicited by visual stimuli from the IAPS. However, they concluded that the effect of acoustic stimuli was often weaker than of visual stimuli by comparing their auditory results to results in other studies examining the effect of visual stimuli on physiological measures. Possible reasons mentioned by Bradley and Lang (2000) for the presumed difference are the specific exemplars of stimuli used (the particular sounds tested may have been less emotional than the particular pictures), effects due to stimulus modality (e.g., more extensive inputs from visual cortex to other areas in the brain compared to auditory) and reasons concerned with the dynamic nature of sounds versus the static nature of images. While a picture is recognized and processed within an instant, information from sound varies over time and needs to accrue in order to be interpreted. However, while these reasons have been suggested why sounds may have a weaker effect than visual stimuli, it has still not unequivocally been proven that such an effect really exists. We here test whether within a single group of observers rather than different groups, sounds, and pictures with approximately equal scores on valence and arousal do indeed differ with respect to their effect on physiological responses. Moreover, we investigate whether elicited emotions and their physiological correlates increase when audio and visual stimuli are combined. Stronger effects could be caused through a type of summation (Nickerson, 1973) or when interaction of the modalities brings the emotional effect to a next level (superadditive effect; Stein and Meredith, 1993). In an fMRI study, Baumgartner et al. (2006b) showed increased activity in emotion processing brain structures when visual emotional stimuli were combined with congruent musical excerpts compared to visual stimuli alone. Consistent with this, subjective and physiological measures in the study by Baumgartner et al. (2006b) reflected more involvement (arousal) for pictures combined with music compared to pictures alone. While these measures did not significantly differ between bimodal and auditory conditions, EEG alpha power measures suggested strongest activation for bimodal compared to the other conditions.

To summarize, in the current study we use blocks of visual, auditory, and bimodal stimuli to induce certain valence and arousal levels. We determine effects of stimulus modality, valence and arousal, as well as their interaction, on heart rate, heart rate variability, and skin conductance without locking variables to stimulus onset. In the following, we present a short overview of the principles behind these dependent variables and how they have been found to be affected by emotional stimuli in previous valence- and arousal-related (stimulus locked) research.

### Cardiovascular measures

Heart rate and its variability are affected by activation and suppression of both the sympathetic and parasympathetic nervous systems. Heart rate variability can be divided along three frequency bands, reflecting three main sources (Mulder, 1988; Veltman and Gaillard, 1998): slow changes (0.02–0.06 Hz) caused by processes like temperature regulation, mid-range changes (0.07–0.14 Hz) related to resonance in the veins caused by the blood pressure regulation, and fast changes (0.15–0.50 Hz) reflecting breathing. Effects of the (rather slow) sympathetic system are visible only in the low and mid frequency bands while effects of the (fast) (Berger et al., 1989) parasympathetic system can be observed in all three bands. Under normal resting conditions, heart rate is carefully adapted to blood pressure such as to keep blood pressure around a certain set point. This adaptation lessens under particular circumstances, such as an increase in mental workload (Mulder, 1980; Aasman et al., 1987), therewith decreasing heart rate variability. Grossman and Taylor (2007) propose that parasympathetically modulated heart rate variability facilitates gas exchange and closely interacts with behavioral, respiratory, and cardiac parasympathetic mechanisms. When the parasympathetic system is suppressed, this adjustment is less tight and heart rate variability decreases. Being affected by both the sympathetic and parasympathetic system, as well as many other physiological processes, associations between heart rate measures and affective reports have been heterogeneous.

With respect to heart rate, recall of both pleasant and unpleasant memories correlate positively with heart rate acceleration (Vrana and Lang, 1990; Cuthbert et al., 2003; Rainville et al., 2006), suggesting that arousal influences heart rate. Using images of the IAPS, Lang et al. (1993) also found a modest positive effect of arousing images on heart rate acceleration, though they reported to not have found this in a previous pilot study. Rather than a positive effect of arousal on heart rate, Ritz et al. (2005) reported heart rate deceleration when scary or happy IAPS pictures were viewed. Also, Bradley and Lang (2000) found that heart rate deceleration was greater when listening to high arousal unpleasant sounds then when listening to low arousal unpleasant sounds. This arousal effect was not seen for pleasant sounds. These three studies aside, most perception studies show valence rather than arousal effects, where pleasant stimuli correlate with higher heart rate acceleration than unpleasant stimuli (Hare et al., 1970; Libby et al., 1973; Winton et al., 1984; Greenwald et al., 1989; Bradley et al., 1990; Lang et al., 1993, 1998; Bradley and Lang, 2000; Anttonen and Surakka, 2005; Codispoti and De Cesarei, 2007; Sokhadze, 2007).

A recent review on studies that examined the association of heart rate variability and work stress concluded that reported work stress is associated with lower heart rate variability (Chandola et al., 2010). Studies on heart rate variability and emotions are mostly dealing with fear or anxiety (George et al., 1989; Friedman and Thayer, 1998; Rao and Yeregani, 2001) where heart rate variability decreases with increased levels of fear. In a study where participants relived emotions, Rainville et al. (2006) found that besides fear, also sadness and happiness decreased high frequency heart rate variability. In contrast to these studies that suggest a negative relation between heart rate variability and arousal, studies in which emotional visual stimuli were used, report increased heart rate variability for erotic images (Ritz et al., 2005) as well as for aversive visual stimuli (Sokhadze, 2007). Whereas studies on mental workload focus their analyses on mid-frequency heart rate variability (reflecting both sympathetic and parasympathetic control), studies on emotions focus on the high frequency band (only parasympathetic).

### Skin conductance

Electrical skin conductance varies with the moisture level of the skin. Since the sweat glands are controlled by the sympathetic part of the autonomous nervous system (Roth, 1983), skin conductance measures can be taken to indicate arousal. Indeed, a large number of studies found an increase in skin conductance with arousal (independent of valence) (Tucker and Williamson, 1984; Winton et al., 1984; Greenwald et al., 1989; Bradley et al., 1990; Tremayne and Barry, 1990, 2001; Cook et al., 1991; Boucsein, 1992, 1999; Barry and Sokolov, 1993; Khalfa et al., 2002). As Table 1 in Chanel et al. (2009) indicates, skin conductance measures are perhaps the most popular physiological signal in studies trying to classify emotional states on the basis of (neuro)physiological signals. Arousal seems more closely associated with increases in skin conductance than heart rate (Barry and Sokolov, 1993; Croft et al., 2004; Wilkes et al., 2010). Skin conductance responses vary with rated arousal in emotional/neutral picture viewing tasks (Lang et al., 1993, 1998; Greenwald et al., 1989).

### Aim and hypotheses

We here aim to manipulate emotional state using blocks of visual, auditory, and bimodal stimuli and determine its effect on physiological responses. Stimuli will be presented in 2-min blocks, corresponding to specific valence and arousal levels. Using variables for which stimulus onset does not need to be known, we examine effects of stimulus modality, valence, and arousal on cardiovascular measures heart rate and heart rate variability as observed over the first minute (1 minute is needed to reliably determine heart rate variability), and on skin conductance over the first half a minute (for skin conductance shorter intervals are sufficient). The remaining duration of the stimulus block is used to examine the course of possible effects over time.

Previous (stimulus locked) research as described in the two sections above lead us to expect that in our study, heart rate will be higher in pleasant stimuli blocks compared to unpleasant blocks, and skin conductance will be higher for high arousing stimulus blocks compared to low arousing blocks. Arousal may affect heart rate variability. We do not expect valence effects on heart rate variability and skin conductance. Valence and arousal effects may be least explicit for auditory stimuli and strongest for bimodal stimuli. This would be reflected by interaction effects between valence and arousal levels and modality.

## Materials and methods

### Participants

Six female and five male participants were recruited through the participant pool of TNO (the research institute where the study was conducted). The participant pool mainly consists of (former) students of a nearby university. Participants were between 20 and 27-years old with a mean age of 23.1 and a standard deviation of 2.0 years. None of them stated to suffer or have suffered from neurological disorders like epilepsy or cerebral hemorrhage, mental illnesses, diabetes, drugs, or alcohol addiction. Participants received a monetary reward to make up for their travel and time. The study is in accordance with the Declaration of Helsinki and has been approved of by the local ethics committee. All participants signed an informed consent form prior to taking part in the experiment.

### Apparatus

Images were displayed on a 19″ Dell 1907FTP LCD screen and audio was presented through Dell AS501 stereo sound bar speakers.

To record ECG, self-adhesive electrodes were attached after having cleaned the contact area with alcohol wipes. The reference electrode was placed on the manubrium of the sternum; the ECG channel electrode was placed at the left, fifth intercostal space; the ECG ground electrode was placed 5–8 cm below the ECG channel electrode. Recording frequency was 512 Hz.

Skin conductance was recorded by custom-made equipment. The fingertips of the index- and middle-finger were attached to steel plate electrodes. A small voltage (0.5 V) was applied across the electrodes and the resultant current flow was recorded at 512 Hz.

### Stimuli

Eight pictures and eight sounds were selected from the IAPS and IADS for each of five emotional blocks. These emotional blocks were unpleasant—low arousal, unpleasant—high arousal, pleasant—low arousal, pleasant—high arousal and neutral—low arousal. Table 1 displays the stimulus numbers of the stimuli used. Table 2 gives the means and standard deviations of the valence and arousal scores as indicated by the IAPS and IADS technical reports (Lang et al., 2005; Bradley and Lang, 2007) for each of the five emotional blocks. Arousal and valence significantly differed between emotional blocks that intended to vary these values (Wilcoxon rank sum tests: all *p*-values < 0.01). Furthermore, the stimuli were selected in such a way that the valence and arousal scores were comparable in value between emotional blocks that correspond in level of valence and arousal (e.g., arousal values were approximately the same in unpleasant—low arousal and pleasant—low arousal blocks as verified by Wilcoxon rank sum tests: all *p*-values > 0.1). In the neutral blocks, arousal was inherently low. The valence and arousal scores of the pictures were comparable to those of sounds, only the valence of the low arousal—unpleasant pictures was lower than the low arousal—unpleasant sounds (Wilcoxon rank sum test *p* = 0.01—all other comparisons > 0.05). For the bimodal condition, an effort was made to pair IAPS and IADS stimuli in the most congruent way as possible (e.g., the “aimed gun” picture was paired to the “gun shot” sound). The combinations of specific visual and auditory stimuli for the bimodal conditions are presented in Table 1.

**Table 1:** **Numbers and names of the visual (IAPS) (Lang et al., 2005) and auditory (IADS) (Bradley and Lang, 2007) stimuli used in each of the five blocks (negative—low arousal, positive—high arousal, negative—high arousal, positive—high arousal, neutral—low arousal)**.

**neg-lowar**		**pos-lowar**		**neg-highar**		**pos-highar**		**neutral-lowar**	
**vis**	**aud**	**vis**	**aud**	**vis**	**aud**	**vis**	**aud**	**vis**	**aud**
2141	242	1463	110	2730	255	4660	202	1390	152
Grieving fem	Female cough	Kittens	Baby	Native boy	Vomit	Erotic couple	Erotic fem	Bees	Tropical
3230	241	2208	813	3060	625	8030	415	1560	114
Dying man	Male cough	Bride	Wedding	Mutilation	May day	Skier	Count down	Hawk	Cattle
3300	280	4623	221	3150	106	8080	352	2220	251
Disabled child	Woman crying	Romance	Male laugh	Mutilation	Growl	Sailing	Sports crowd	Male face	Nose blow
8230	283	5910	601	6250	289	8180	353	2635	113
Boxer	Fight	Fireworks	Colonial music	Aimed gun	Gun shot	Cliff divers	Base ball	Cowboy	Cows
9120	611	7330	721	9050	501	8185	360	3210	729
Oil fires	Battle taps	Ice cream	Beer	Plane crash	Plane crash	Sky divers	Roller coaster	Surgery	Paper
9520	423	8040	816	9250	600	8200	311	4613	364
Kids	Injury	Diver	Guitar	War victim	Bike wreck	Water skier	Crowd	Condom	Bar
9611	699	8120	820	9252	711	8400	365	7620	410
Plane crash	Bomb	Athlete	Funk music	Dead body	Siren	Rafters	Party	Jet	Helicopter
9301	250	8496	220	9921	244	8501	367	9411	722
Toilet	Male sneeze	Water slide	Boy laugh	Fire	Man wheeze	Money	Casino	Boy	Walking

Stimuli that were presented simultaneously in the bimodal condition are in the same row of each block.

**Table 2:** **Valence and arousal scores with their standard deviations of stimuli used in each of the five emotional blocks as previously reported in the IAPS (Lang et al., 2005) and IADS (Bradley and Lang, 2007) documentation and as currently reported by our participants**.

	**Valence**				**Arousal**			
	**Score**		***SD***		**Score**		***SD***	
	**Prev**	**Curr**	**Prev**	**Curr**	**Prev**	**Curr**	**Prev**	**Curr**
**VISUAL BLOCK**								
neg-lowar	2.60	2.35	1.66	0.71	5.39	4.63	2.20	0.89
pos-lowar	7.33	7.48	1.59	0.64	5.44	5.33	2.30	1.02
neg-highar	2.29	1.84	1.65	0.54	6.64	6.23	2.15	1.20
pos-highar	7.47	7.13	1.58	0.53	6.75	5.86	2.08	1.15
neutral	5.12	4.85	1.74	0.50	5.06	4.35	2.00	0.81
**AUDITORY BLOCK**								
neg-lowar	3.18	3.17	1.81	0.40	5.68	4.82	1.81	0.80
pos-lowar	6.98	7.07	1.86	0.64	5.61	5.44	1.86	0.98
neg-highar	2.73	2.92	1.68	0.55	6.80	5.40	1.68	1.01
pos-highar	7.09	6.73	1.84	0.64	6.88	5.94	1.84	1.05
neutral	4.88	5.26	1.76	0.43	5.48	4.05	1.76	1.10
	**Valence**				**Arousal**			
	**Score**		***SD***		**Score**		***SD***	
**BIMODAL BLOCK**								
neg-lowar	2.30		0.53		5.05		0.98	
pos-lowar	7.48		0.71		5.70		1.14	
neg-highar	2.05		0.51		6.13		0.89	
pos-highar	7.14		0.62		5.99		0.79	
Neutral	4.68		0.53		4.16		1.21	

For the bimodal block, we only present valence and arousal scores from our own participants.

### Design and procedure

Participants were tested individually in the laboratory. Participants were asked to sit still, and to watch and listen to the stimuli. Stimuli were presented in three modality blocks (visual, auditory, and bimodal). The order of these blocks was picked randomly for each participant. The modality blocks were separated by a 15-s rest period. Each modality block consisted of five emotional blocks (unpleasant—low arousal, unpleasant—high arousal, pleasant—low arousal, pleasant—high arousal and neutral—low arousal), in randomized order. The emotional blocks were separated by a 15-s rest period. Each emotional block consisted of two repetitions of 8 stimuli, in randomized order. Each stimulus was presented for 6 s. Stimuli were separated by blank screens with a jittered duration (average duration 1500 ms). After the stimulus presentation and physiological recordings were over, participants rated the stimuli they had observed on a SAM rating scale for arousal and valence [as used in Lang et al. (2005), Bradley and Lang (2007)]. This was done by presenting them with the stimuli again on a laptop, separately for each modality block.

### Signal processing

The ECG signal was filtered by a 2–200 Hz band-pass 2-sided Butterworth filter. For each participant, we then determined the median RRI of the first 60 s of each of the 15 stimulus blocks (3 modality × 5 emotional blocks). RRI is the interval between successive heart beats or more precisely, the interval between subsequent R-peaks in the ECG. The peak detection algorithm used to identify these peaks required an R-peak to occur at least 222 ms after the previous one (corresponding to a maximum allowed heart rate of 270 b/m). The first R-peak in an ECG trace needed to be between 1 and 5 mV (as measured between the R-peak and the subsequent S-valley) while subsequent peaks were identified as such if they crossed a threshold starting at the height of the just identified peak and then exponentially decreasing over time to an asymptote of 1 mV. This procedure proved to reliably detect heart beats as indicated by visual inspection of the raw ECG signal with labeled peaks. The inverse of the RRIs provided us with the heart rate. Heart rate variability was computed as the root mean squared successive difference (RMSSD: Goedhart et al., 2007) between the RRIs, normalized by dividing this value by the mean RRI. This measure reflects high frequency heart rate variability.

The skin conductance signal was filtered by a 30 Hz low-pass 2-sided Butterworth filter. For each participant, we determined skin conductance over the first 30 s of each of the 15 stimulus blocks by averaging the inverse of the recorded signal. Subsequently, each value was baselined by subtracting skin conductance averaged over the 10 s preceding the block (i.e., during rest).

In order to indicate the course of the different effects over time, we repeated the analyses of ECG and skin conductance over different time intervals: for ECG also the second half of each block was analyzed and for skin conductance also the second, third and fourth quarter.

### Statistical analysis

For each dependent variable (heart rate, RMSSD, and skin conductance), a repeated measure ANOVA was performed on data from the unpleasant—low arousal, unpleasant—high arousal, pleasant—low arousal, pleasant—high arousal blocks with modality (3 levels), arousal (2 levels) and valence (2 levels) as independent variables. We chose an alpha level of 0.05.

For the variables that were significantly affected by arousal and/or valence, we computed the size of the effect for different time windows for each participant, averaged over modality (because modality turned out not to affect the responses). This was done by subtracting the value of the variable during low arousal of that during high arousal, and the same for low and high valence. To test for changes of these differences over time, we compared the differences between the first and the last time window using paired *t*-tests.

Data from the neutral conditions were not analyzed here.

## Results

### Ratings

We analyzed the ratings as provided by our participants for the visual and auditory stimuli in the same way as we analyzed the previously reported ratings by Lang et al. (2005) and Bradley and Lang (2007) when constructing the stimulus set (section Stimuli).

Valence significantly differed between emotional blocks that were intended to vary these values (Wilcoxon rank sum tests: all *p*-values < 0.01). High arousal and low arousal low valence images significantly differed in arousal (*p* < 0.01). However, the difference between high and low arousal failed to reach significance for high valence images, low valence sounds and high valence sounds. Still, effects of arousal were found on the physiological measures as described in section Effects of Modality, Valence and Arousal on Physiological Variables and Arousal and Valence Effects Over Time.

Arousal did not differ between emotional blocks that were intended to induce equal arousal, both within and between modalities. Consistent with our intention, valence scores did not differ for all high valence emotional blocks, within and between modalities. However, unpleasant high arousal images were judged as lower in valence than unpleasant low arousal images (*p* = 0.02). Also, unpleasant images were rated as lower in valence than unpleasant sounds (*p* = 0.02 for low arousal, and *p* < 0.01 for high arousal). Still, we did not find effects including modality on the physiological measures as described in section Effects of Modality, Valence and Arousal on Physiological Variablesand Arousal and Valence Effects Over Time.

Bimodal stimuli did not systematically show more extreme arousal and valence ratings compared to unimodal stimuli. Only for low arousal blocks, the difference in valence between pleasant and unpleasant stimuli tends to be larger for bimodal stimuli compared to either unimodal condition: an average 5.18 difference in bimodal valence scores versus 5.13 for the second largest valence difference which was found for the visual condition. However, a Wilcoxon rank sum test on these differences did not indicate a significant modality effect.

Note that differences between the ratings of our participants and the ones in the studies by Lang et al. (2005) and Bradley and Lang (2007) can be caused by a difference in rating methodology. Whereas Lang and colleagues asked their participants to rate their experienced emotion immediately after stimulus presentation, we presented our participants with the stimuli again at the end of the experiment in order to perform the rating. We modified the rating procedure such as to not disturb the emotion that we intended to elicit through the blocks of stimuli.

### Effects of modality, valence, and arousal on physiological variables

Due to technical problems, ECG data were missing for one subject in two conditions and skin conductance data were completely missing for another subject. Data of these subjects are left out in respectively ECG and skin conductance analyses.

**Figure 1:**
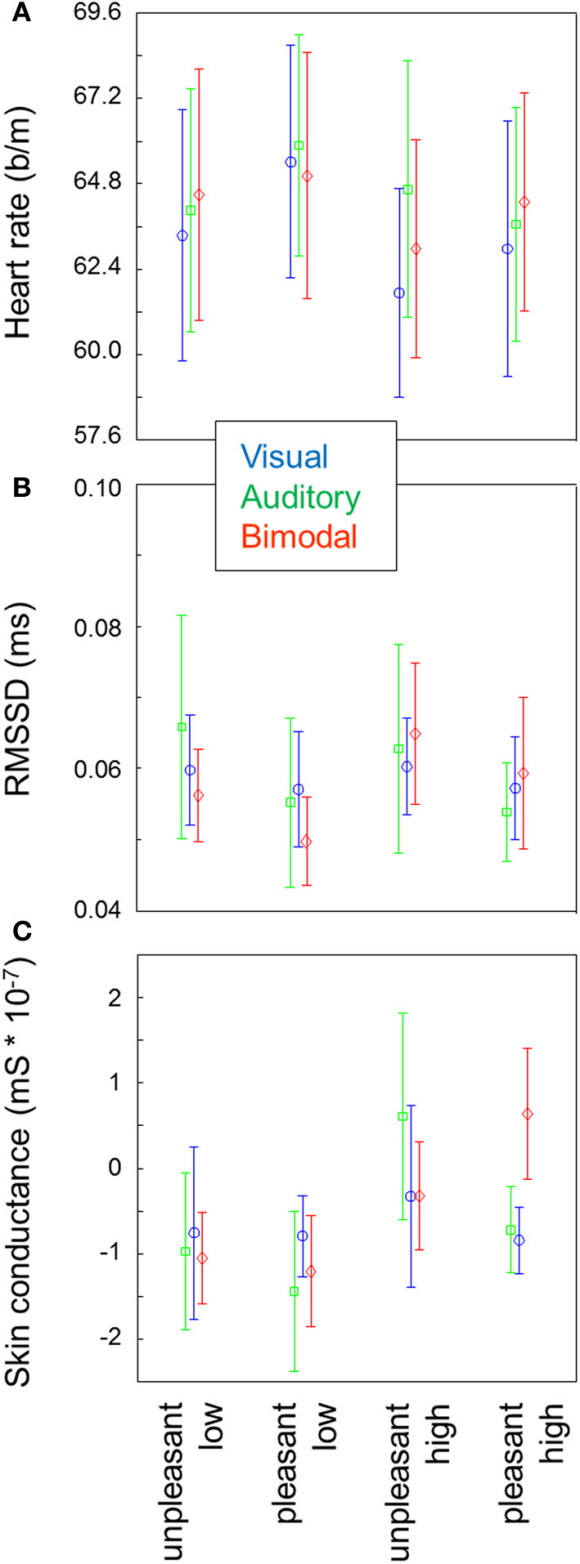
**Heart rate (A), RMSSD, (B) and skin conductance (C) for each of the conditions.** Error bars represent standard errors of the mean between participants. For heart rate, a repeated measure ANOVA indicates main effects of arousal and valence. For heart rate variability and skin conductance, repeated measures ANOVAs indicate main effects of arousal.

Figure 1A shows the average heart rate for each of the 15 conditions. Heart rate was lower for high arousal compared to low arousal stimuli [*F*_(1, 9)_ = 25.66, *p* < 0.01], and higher for pleasant stimuli compared to unpleasant stimuli [*F*_(1, 9)_ = 5.59, *p* = 0.04]. There was no effect of modality and no 2- or 3-way interactions between arousal, valence and modality.

Heart rate variability as operationalized by normalized RMSSD was exclusively affected by arousal [*F*_(1, 9)_ = 5.94, *p* = 0.04] where higher variability was found in the high arousal blocks compared to the low arousal blocks (Figure 1B). No other main or interaction effects approached significance.

Skin conductance increased with arousal [*F*_(1, 9)_ = 11.83, *p* < 0.01; Figure 1C]. No other main or interaction effects approached significance.

### Arousal and valence effects over time

**Figure 2:**
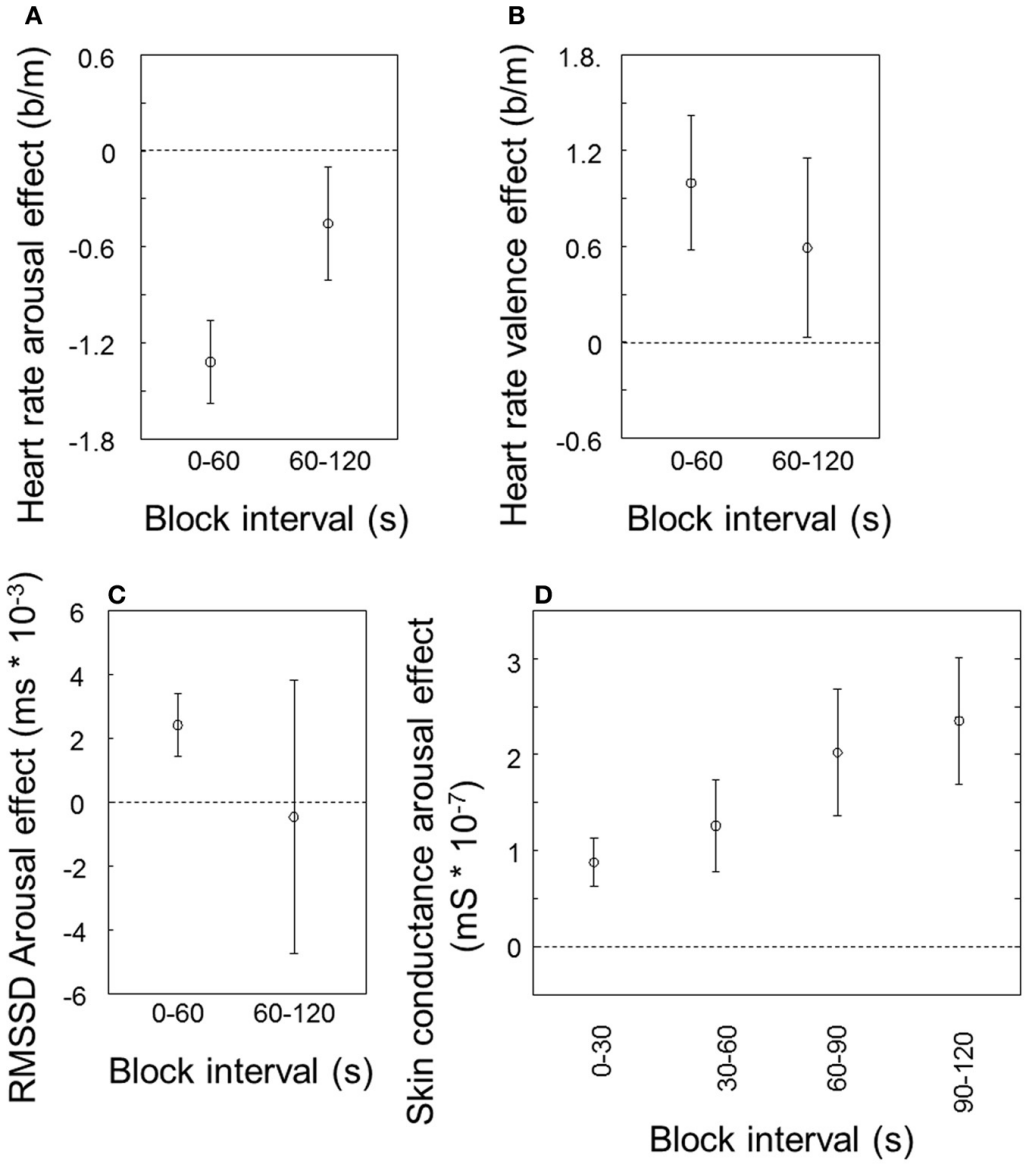
**Arousal and valence effects over time, averaged over modalities.** Arousal effect on heart rate **(A)**, valence effect on heart rate **(B)** and arousal effect on heart rate variability (RMSSD) **(C)**, for the first and second 60 s of the block; arousal effect on skin conductance for the first through fourth 30 s of the block **(D)**. Error bars represent standard errors of the mean between participants.

Figure 2 indicates how the effects of valence and arousal as reported above change over time. The effects of arousal and valence on heart rate (Figures 2A and B, respectively) tend to be smaller during the second half of the stimulus blocks compared to the first half, only significantly so for arousal [*t*_(9)_ = 2.27, *p* = 0.049]. The effect of arousal on RMSSD (Figure 2C), visible for the first half of the block, has on average disappeared in the second half and dramatically increased in variability between participants. A paired *t*-test does not indicate a significant difference between the first and the second half. In contrast to the cardiovascular measures, the effect of arousal on skin conductance becomes stronger rather than (a trend to) weaker (Figure 2D; *t*_(9)_ = 3.06, *p* = 0.01).

### Effect of outliers

In order to check for possible effects of outlying values, we identified blocks that differed for more than 3 standard deviations from the mean for heart rate, RMSSD, and skin conductance. There were no outliers for heart rate. For skin conductance, two participants had one or more outlying emotional blocks, and for RMSSD this was the case for one participant. Excluding these participants respectively from the skin conductance and RMSSD analyses as reported in Arousal and Valence Effects Over Time and did not change the pattern of significant results, indicating that our results cannot be explained by sample outliers.

## Discussion

We successfully manipulated our observers' emotional state by presenting visual, auditory and bimodal stimuli in blocks, as indicated by effects of valence and arousal on different physiological variables. This was found without locking variables to stimulus onset and despite a large variability in the overall values of the physiological variables between participants (indicated by the large error bars in Figure 1). One physiological variable that has rarely been used in previous emotional perception research, heart rate variability, displayed an effect of arousal. Heart rate variability and skin conductance increased with arousal. Heart rate decreased with arousal and was higher for pleasant stimuli compared to unpleasant stimuli. Over two minute intervals, cardiovascular effects habituated or tended to habituate whereas the effect on skin conductance increased. We did not find any effect of stimulus modality. We discuss each of these findings below.

### Heart rate

As described in the Introduction, perception studies that examined the effect of arousal on heart rate produced mixed results: a positive effect (Lang et al., 1993), a negative effect (for unpleasant stimuli—Bradley and Lang, 2000) and no effect (no loading on arousal as indicated by factor analyses—Lang et al., 1998). Studies on recalling emotional (versus neutral) memories generally show increasing heart rate with arousal (Vrana and Lang, 1990; Cuthbert et al., 2003; Rainville et al., 2006). We here find heart rate to decrease with arousal. Generally, heart rate may be expected to increase with arousal in order to get the body ready for action. However, in studies where participants only observe emotional stimuli or events (like ours) another process may dominate. Previous studies showed that allocating attentional resources to a perceived stimulus elicits heart rate deceleration over the first few seconds after stimulus onset (Lacey and Lacey, 1970; Graham, 1992; Codispoti et al., 2001). High arousal probably causes increased information processing or attention and thus a larger drop in heart rate.

The lower heart rate when unpleasant stimuli were presented compared to pleasant stimuli is consistent with the large majority of the perception literature (Hare et al., 1970; Libby et al., 1973; Winton et al., 1984; Greenwald et al., 1989; Bradley et al., 1990; Lang et al., 1993, 1998; Bradley and Lang, 2000; Anttonen and Surakka, 2005; Codispoti and De Cesarei, 2007; Sokhadze, 2007) though there are a few exceptions (Ritz et al., 2005; Dimberg and Thunberg, 2007). It should be noted though that this valence effect on heart rate is not a general finding when looking at the emotion literature as a whole. In her literature review, Kreibig (2010) showed that the effect of valence on heart rate can be in both directions. As with the effect of arousal on heart rate, the type of emotional stimuli seems crucial. Kreibig proposes that an important distinguishing factor is passivity. Emotions involving an element of passivity (e.g. non-crying sadness, contentment) rather result in heart rate decrease in contrast to more “active” emotions (joy, anger). In this vein, one could speculate that unpleasant pictures elicit sadness rather than anger, and pleasant pictures elicit joy rather than contentment resulting in the valence effect on heart rate as reported in the perception literature as well as in the present study.

### Heart rate variability

Most studies on perception of emotional stimuli do not report heart rate variability because physiological variables are analyzed over short time intervals and locked to stimuli. Two studies that did, reported increased high frequency heart rate variability for erotic (Ritz et al., 2005) and aversive (Sokhadze, 2007) images. We also found an increase of heart rate variability with arousal. In contrast, heart rate variability is reported to decrease with arousal in the fields of stress (Chandola et al., 2010) and fear or anxiety (George et al., 1989; Friedman and Thayer, 1998; Rao and Yeregani, 2001). As with heart rate, this difference may be related to the type of stimuli used and with it, the required action and the exact quality of the experienced emotion. Another factor that may be important here is breathing frequency. Slow, deep breathing produces a sharp increase in heart rate variability (Angelone and Coulter, 1964; Grossman and Taylor, 2007). In our study, participants may have taken a few deep breaths during high and not during low arousal conditions, explaining the high heart rate variability for high arousal. Ritz et al. (2005) showed that emotional pictures can indeed differentially influence respiration. However, they corrected for this in their measure of high frequency heart rate variability and still found an increase in variability with (erotic) arousal pictures.

### Skin conductance

Our finding that skin conductance increased with stimulus arousal is well in line with results described in the literature (Tucker and Williamson, 1984; Winton et al., 1984; Greenwald et al., 1989; Bradley et al., 1990; Tremayne and Barry, 1990, 2001; Cook et al., 1991; Boucsein, 1992, 1999; Barry and Sokolov, 1993; Khalfa et al., 2002). Several authors stated that arousal is more closely connected to skin conductance than to heart rate (Barry and Sokolov, 1993; Croft et al., 2004; Wilkes et al., 2010). The positive effect of arousal on skin conductance in our study suggests that our stimuli, despite the decrease in heart rate and the increase in heart rate variability, indeed induced different arousal levels as intended.

### Effects of emotional stimuli over longer intervals

We found that over two minute intervals, valence and arousal effects in cardiovascular measures (tend to) habituate, whereas the effect of arousal on skin conductance increases. Parasympathetic effects are faster (in the order of milliseconds) than sympathetic effects (in the order of seconds; Rainville et al., 2006; Grossman and Taylor, 2007). However, it is unlikely that this can explain the difference between the courses of the effects over minutes. Another reason might be that while sweat glands are controlled by the sympathetic system, the heart is innervated by both sympathetic and parasympathetic systems, therewith possessing a “brake” that the sweat glands lack. Finally, building a layer of sweat is a relatively slow process. For stimulus locked variables and image blocks of several minutes in duration, Bradley et al. (1996) reported a constant image valence effect on skin conductance and heart rate.

### Modalities

Recording responses within a single group of participants, we did not find weaker effects for auditory stimuli compared to visual stimuli as suggested by Bradley and Lang (2000)—even though our match of auditory and visual stimuli was not perfect (see section Stimuli). For heart rate, a variable that Bradley and Lang (2000) specifically reported to be affected relatively little by emotional sounds, there was a trend for visual stimuli to exert the strongest effects (arousal and valence; Figure 1A) but it was not close to significance. Bimodal stimuli did not enhance arousal or valence effects over unimodal stimuli; only in heart rate variability (Figure 1B) the expected trend was found, but again, this was far from significant. Note that our participants also did not rate bimodal stimuli more extremely in valence and arousal than unimodal stimuli. Possibly, including more participants would have resulted in (significant) modality effects but at least, our findings show that potential modality effects are weak. In our experiment, bimodal presentation may not have enhanced emotion because the stimuli were not optimally congruent; though not completely off, in most cases they clearly did not originate from the same source. Baumgartner et al. (2006a) used musical stimuli to induce emotions. While music is less comparable to IAPS pictures than IADS sounds, the advantage of music over sounds is that it can more easily be combined with pictures to produce bimodal stimuli that can be considered congruent. Still, Baumgartner and colleagues also did not find stronger involvement (arousal) as reflected by subjective and physiological measures for bimodal stimuli compared to auditory stimuli alone.

## Conclusion

In line with Bradley et al. (1996), our results strongly suggest that sustained emotions can be elicited by repeatedly presenting visual and auditory stimuli of similar arousal and valence over time intervals of at least two minutes. This provides a tool for emotion manipulation in emotion research and possibly in treatment or training situations, e.g., where individuals need to learn to optimally function during the experience of negative emotions. Care must be taken not to simply generalize results of studies using passive perception of emotional stimuli to situations with (other) emotional stimuli that elicit (other) actions than the ones being studied. Another limitation is our relatively small sample size. We measured effects of valence and arousal using physiological variables that were not locked to stimulus onset. This is practical and necessary for measuring emotions in real life situations where there are no specific stimuli to lock responses to or where the exact time of stimulus onset is unknown, such as when evaluating valence or arousal elicited during human-machine interaction or advertisement movies. We found that valence and arousal effects of real life images on heart rate, heart rate variability and skin conductance do not differ from the effects of real life sounds. This finding suggests that emotional auditory stimuli can be used in situations where visual presentation is less practical (e.g., when the visual sensory channel is already occupied). Presenting visual and auditory stimuli together does not enhance the effects. Possibly effects can be enhanced when bimodal stimuli are more congruent, i.e., clearly originate from the same source.

### Conflict of interest statement

The authors declare that the research was conducted in the absence of any commercial or financial relationships that could be construed as a potential conflict of interest.
